# Systems 1 and 2 thinking processes and cognitive reflection testing in medical students

**Published:** 2016-10-18

**Authors:** Shu Wen Tay, Paul Ryan, C Anthony Ryan

**Affiliations:** 1 Department of Neonatology, Cork University Maternity Hospital, Ireland; 2 Department of Paediatrics and Child Health, University College Cork, Ireland; 3 Teagasc, Moorepark, Fermoy, Co. Cork, Ireland

## Abstract

**Background:**

Diagnostic decision-making is made through a combination of Systems 1 (intuition or pattern-recognition) and Systems 2 (analytic) thinking. The purpose of this study was to use the Cognitive Reflection Test (CRT) to evaluate and compare the level of Systems 1 and 2 thinking among medical students in pre-clinical and clinical programs.

**Methods:**

The CRT is a three-question test designed to measure the ability of respondents to activate metacognitive processes and switch to System 2 (analytic) thinking where System 1 (intuitive) thinking would lead them astray. Each CRT question has a correct analytical (System 2) answer and an incorrect intuitive (System 1) answer. A group of medical students in Years 2 & 3 (pre-clinical) and Years 4 (in clinical practice) of a 5-year medical degree were studied.

**Results:**

Ten percent (13/128) of students had the intuitive answers to the three questions (suggesting they generally relied on System 1 thinking) while almost half (44%) answered all three correctly (indicating full analytical, System 2 thinking). Only 3–13% had incorrect answers (i.e. that were neither the analytical nor the intuitive responses). Non-native English speaking students (n = 11) had a lower mean number of correct answers compared to native English speakers (n = 117: 1.0 s 2.12 respectfully: p < 0.01). As students progressed through questions 1 to 3, the percentage of correct System 2 answers increased and the percentage of intuitive answers decreased in both the pre-clinical and clinical students.

**Conclusions:**

Up to half of the medical students demonstrated full or partial reliance on System 1 (intuitive) thinking in response to these analytical questions. While their CRT performance has no claims to make as to their future expertise as clinicians, the test may be used in helping students to understand the importance of awareness and regulation of their thinking processes in clinical practice.

## Introduction

Making a diagnosis is central to medical practice. A correct diagnosis sets off a chain of events, investigations, and therapeutic treatments, that lead to appropriate management. This is done through clinical reasoning, the “cognitive process that is necessary to evaluate and manage a patient’s medical problem.”^1^ Some experts estimate that 75% of diagnostic failures can be attributed to clinician diagnostic thinking failure from multiple causes including inadequate knowledge, faulty data gathering, and/or faulty verification.^2^ Thus, the clinician’s ability to provide safe, high-quality care is dependent upon their ability to reason, think, and judge.

Despite the importance placed on patient safety in the modern curriculum^3^, medical education at present has built an environment that does not always actively promote development of clinical reasoning. Educators recognize its importance in developing expertise, but it is often not an explicit educational objective.^4^ Part of this is due to the belief that clinical reasoning will be acquired on its own over time with practice and an accumulation of knowledge.^5^ Norman and Eva^6^ in a systematic review of the literature, concluded that strategies directed at encouraging both analytical and non-analytical reasoning could lead to some gains in diagnostic accuracy. Thus, knowing how doctors think, make decisions, and make errors in thinking is important for novice and expert clinical decision makers, but also for educators who will need to have multiple strategies to teach both analytical and non-analytical reasoning.^7^

Decision-making is complex. It is partly based on the dual-process theory of Epstein and Hammond,^8^ recently popularized in Daniel Kahneman’s book “Thinking Fast and Slow.”^10^ Two families of cognitive operations, called System 1 (intuitive) and System 2 (analytical) thinking, are used in decision-making. System 1 thinking is often described as a reflex system, which is “intuitive” and “experiential” or “pattern recognition”, which triggers an automated mode of thinking. It is generated without much conscious effort and channels the available information through a subconscious pattern recognition based on similar past situations;^11^,^12^ this is often described as the “gut feeling”. When problems are routine and when under time constraint, System 1 kicks in. When an individual is more dependent on System 1 thinking (for example, HALT: “hungry, angry, tired or late” or under conditions of illness, substance abuse or emotional distress), the accuracy of decision-making can be adversely affected.^14^ Nevertheless, there is evidence that System 1 thinking is an indispensable element of clinical decision-making in physician primary care.^15^,^16^ Although System 2 (analytical) thinking is more deliberate than System 1, the latter is not necessarily less capable. On the contrary, complex cognitive operations eventually migrate from System 2 to System 1 (i.e. become more automatic) as proficiency and skill are acquired and pattern matching has replaced effortful serial processing.

System 2 is the more “analytical,” “deliberate” and “rational” side to the thinking process. It is pieced together by logical judgment and a mental search for additional information acquired through past learning and experience.^17^,^18^ The data are then processed carefully, through a conscious application of rules, making it a much slower and cognitively demanding process but more likely to lead to better decisions. The analytical system is engaged usually when there is uncertainty, complexity, or the outcomes give little room for error but there is time to think.^19^,^20^ System 2 thinking is slow, requiring significant cognitive effort, and, though it is less prone to error, is not foolproof.

Experts, drawing upon greater quantities of information within their field, are occasionally subject to cognitive errors and biases, by picking up the wrong information or “distracting cues”, resulting in diagnostic errors. When used alone, System 2 thinking can lead to poorer performance by slowing action processes down. Experience, despite being a yardstick of the expert, does not necessarily translate into better performance. Indeed, experience, without feedback or reflection, can often be the fertile ground for the development of faulty thinking.^21^,^22^

Thus, Systems 1 and 2 thinking are useful in the right place and the right time; indeed, they complement each other. Taken together, they promote greater efficiency in thinking, decision-making and action, and help bring order to chaos and uncertainty.^23^ Whether to use Systems 1 or 2 thinking in a given clinical situation depends on the complexity of the situation in relation to the individual’s capabilities, past experiences, and self-confidence.

The Cognitive Reflection Test (CRT) is a three-question test designed to measure respondent’s ability to activate metacognitive processes allowing them to switch to System 2 thinking. In other words, it is the disposition to resist reporting the response that first comes to mind.^24^ As explained by the CRT inventor Shane Frederick: “The three items on the CRT are “easy” in the sense that their solution is easily understood when explained, yet reaching the correct answer often requires the suppression of an erroneous answer that springs “impulsively” to mind.” In his study, Frederick has shown a reduction in intuitive answers as the questions precede from question 1 to 3 and also a gender bias with better performance among the male population.^24^ The CRT has also been used in a group of judges in the United States.^25^ Judges are thought to be predominantly intuitive thinkers, picking up on intuitive clues that lead them to reach conclusions that they later rationalize. This study showed that only two thirds of the judges gave the right (deliberative) answer to one or more of the three CRT questions, confirming a significant reliance on System 1 (intuitive) thinking in the remaining responses. Of course, these results are not generalizable as to how judges think in courtroom practice. Nevertheless, the CRT may have been a useful exercise in encouraging the judges to be aware of and to regulate their thinking, in particular metacognition, the executive function that turns on their System 2 thinking that can, among other things, expose their cognitive biases.

The CRT has not, to our knowledge, been tested in the medical profession. The aim of this study was to evaluate the level of Systems 1 and 2 thinking in medical students using the CRT. Since the development of clinical expertise is often associated with more automatic System 1 thinking, we also wanted to compare the CRT responses of students in clinical practice (“experts”) and those in pre-clinical years (“novices”). We also wished to test the question progression improvement phenomenon and the possible gender bias seen above.^21^ Although not a specific aim of this study, we believe the CRT test, when used with students, can help them understand the differences between intuitive and analytical thinking in decision making in clinical practice.

## Methods

The assessment tool used in this study was the internationally validated CRT, originally developed as a measure of a type of cognitive ability.^24^ The questions, along with the intuitive (incorrect) and the analytical (correct) answers, are as follows:

CRT question 1: This question required respondents to evaluate the cost of a ball given that the total cost of a bat and a ball was $1.10 and the bat cost $1.00 more than the ball. An intuitive (impulsive) answer that the ball costs $0.10 does spring to mind by subtracting $1.00 from $1.10. However, should this be the case, the total cost of the bat and ball would be $1.20, which is incorrect. Hence, the right answer is $0.05.
CRT question 2: This question asks respondents to evaluate, if 5 machines take 5 minutes to make 5 widgets, how long does is take 100 machines to make 100 widgets. Again, the impulsive answer that springs to mind is 100 minutes, but if one were to take a step back and consider, it would take 1 machine 5 minutes to make 1 widget. Therefore, it would take 100 machines 5 minutes to make 100 widgets.
CRT question 3: This question gave the background of a lily patch in a pond. Each day the lily patch doubled in size. It takes 48 days for the lily patch to cover the entire pond and respondents are asked how long it would take for the lily pad to cover half the pond. The intuitive answer would be to take the half of 48 (day 24), but logically, if the patch were to double in size every day, the day before it covers the entire lake it would cover half the lake (day 47).

Thus the correct answers are, in summary, 5, 5 and 47, while the intuitive answers are 10, 100 and 24, respectfully.

After obtaining ethical approval from Research Ethics Committee of the Cork Teaching Hospitals, medical students in the School of Medicine, University College Cork, from Years 2 & 3 (pre-clinical), and Years 4 (in clinical practice) of a 5-year Medical Degree course were approached to participate in this study. The CRT was distributed to students at the end of a lecture, thus allowing them 10–15 minutes to complete the CRT, in addition to a few demographic questions. A voluntarily completed response was taken to indicate consent for participation. Respondents were assured that participation was anonymous and would have no bearing on their future medical education. Statistical analysis was by student *t*-test for continuous data and Spearman rank correlation to test the association between ranked variables.

## Results

There were approximately 90 students in the pre-clinical and 90 students in clinical class, i.e. 180 students in total, of whom 130 (72%) completed the survey. Two students had previously been exposed to the CRT and were excluded. Of the remaining 128 students, 49 were male and 79 were female, while 61 were pre-clinical and 67 were clinical students. Ten percent of students (13/128) answered none of the CRT questions correctly, 21% (27/128) answered one correctly, 25% (32/128) answered two questions correctly while 44% (56/128) answered all three correctly. Over half of respondents (56%) obtained the correct (analytical) answer to the first question, with 40% giving the intuitive answer. More than two thirds of respondents (70%) got the second question right, with 22% getting the intuitive answer. For question 3, 77% of respondents got the right answer, with 14% getting the intuitive answer. The mean number of questions answered correctly was 2.02. The students returned a total of 388 questions: 67% (259) were correct answers, 25% (97) were intuitive answers and 8% (32) were incorrect answers.

**Figure 1:**
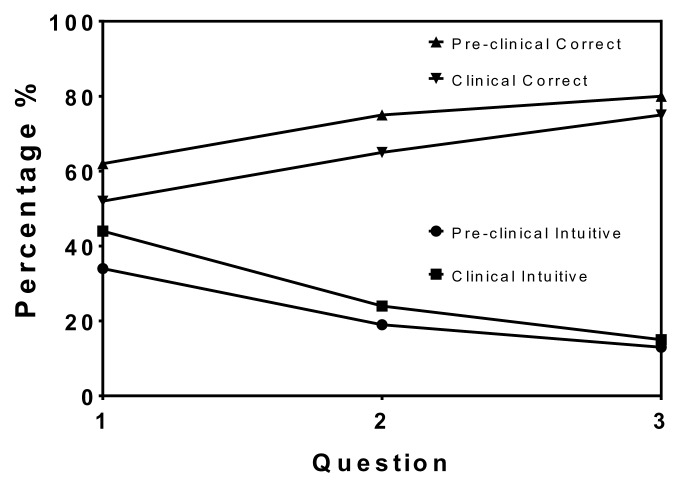
Correct answers increased and intuitive answers decreased in both pre-clinical and clinical students as they progressed from question 1 to 3

The outcomes from the individual three questions, by pre-clinical and clinical students, are presented in Table 1 and Figure 1. The percentage of correct answers increased as the questions progressed from question 1 to 3. At the same time, the percentage of intuitive answers decreased, while incorrect responses ranged between 3 to 13%.

Pre-clinical respondents gave 5–10% more correct and 2–10% less intuitive answers than their clinical counterparts for each question (Figure 1). However, pairwise analysis of the means showed no significant differences between pre-clinical and clinical respondents.

Approximately 9% (11/128) of respondents were international students who did not have English as a childhood language. There was a significant difference between the mean number of correct answers of students who had English as a childhood language (2.12, *n* = 117) and those who did not (1.0, *n* = 11: t test; *p* < 0.01). Conversely, respondents who were non-native English speakers gave significantly more intuitive answers (1.6, *n* = 11) than English speakers (0.7, *n* = 117: t test; *p* < 0.01). There was no relationship between age of respondents, or gender of respondents (male n = 49 and female *n* = 79) and correct (72% vs 65%), intuitive (23% vs 27%) or incorrect (5% vs 8%) answers, respectively.

## Discussion

The CRT is designed to measure the ability of respondents to activate thinking processes that switch to System 2 thinking where System 1 (more intuitive) thinking might lead them astray. To our knowledge, this is the first study to test the CRT on medical students. Our study confirmed that less than half (44%) of the medical students answered all three questions correctly (i.e. were fully metacognitive in engaging System 2 thinking) while one in 10 students answered none of the questions right (suggesting they did not think metacognitively to engage System 2 thinking and generally relied on intuitive thinking). Thus, more than half of the students demonstrated full or partial reliance on intuitive thinking in responding to these analytical questions.

A minority of students (< 13%) had incorrect answers that were neither the analytical nor the intuitive responses. It is possible that these students may have recognized they should switch from System 1 thinking (i.e. they activated some metacognitive processes), but were unsuccessful in their System 2 thinking. Using focus groups to ask participants to explain the manner in which they went about solving each question would have answered this conundrum. However, this was outside the scope of the current study but a useful direction for further research. Finally, a small number of students in this study did not have English as a childhood language and had lower correct responses. A lower level of functioning in the English language may have affected their score due to a less accurate sense of the situations being described in the problems.

As was seen in Frederick’s studies and also in the present study, System 2 responses increased and System 1 answers decreased with progression through the CRT questions.^24^ According to Frederick, the first question is commonly regarded as the easiest and the third, the hardest. Thus, when confronted with problems of a harder nature, respondents use their System 2 processes to override their System 1 intuitive processes to obtain the correct answer. Fredrick found that men scored higher than women on the CRT. He postulated that men, supposedly having more learned skills in mathematics, were less likely to go with the intuitive responses.^24^ We found no gender differences in the CRT scores in our study; however, it was not powered enough to show a significant difference if one existed.

The CRT is a test of cognition and care must be taken not to interpret these results as an index of the medical students’ current or future clinical reasoning. Performance on a math problem has relatively low stakes compared with health care decision-making. Low scoring students may have faulty mathematical intuition based on the CRT, but there is no evidence as yet to say they have faulty intuition in general, particularly medical intuition. It may be of interest in a future study to link CRT responses to subsequent clinical decision-making. However, it is most unlikely that a single mathematical examination such as the CRT could predict future performance in clinical reasoning and judgment. Instead, as it stands, we believe the test can be used to help students understand the differences between analytic and intuitive thinking, the importance of both systems thinking, and especially the need to develop their metacognitive skills. In addition, using the CRT and answering the questions correctly, has been shown to activate System 2 processes and may help prepare students for metacognitive thinking.^26^

The objective of the present study was not to make correlations or reach conclusions that mathematical reasoning predicts or facilitates diagnostic decision-making. However, we observed that students in pre-clinical years demonstrated some evidence of more cognitive override (metacognition) than students in clinical practice although this was not statistically significant. The intuitive answers for the CRT mathematical problems were intrinsically incorrect. In medical practice, intuitive responses are not always wrong. In their work on intuition Tracy et al^15^ stated that: “There was overwhelming agreement that intuition plays a vital role in the practice of family medicine” and that “intuition has its origins in personal clinical experience.” Intuition may also be adaptive in complex situations where decisions are required in a timely fashion; for instance, intuitive responses are essential in emergency situations. Nevertheless, where possible, intuition should be guided and formed by System 2 thinking to reduce the possibility of error or cognitive biases.

The CRT has previously been used at an educational session at the Florida Conference of Circuit Judges in 2006.^25^ The medical students in the present study scored higher than the judges, correctly answering a mean of 2.02 of the CRT questions compared to 1.23 for the judges. The judges were also more likely to respond with intuitive responses, in that only two thirds of the judges (compared to 90% of the medical students), gave the right (deliberative) answer to one or more of the CRT questions. There were a number of differences between the studies, however, that caution direct comparisons. The CRT questions in the Judges’ study were embedded within a larger questionnaire that was administered over 45 minutes. In contrast, the medical student questionnaire consisted of the 3 CRT questions and a number of demographic questions that was administered over 10–15 minutes between lecture slots.

There were a number of limitations in this study. It was only possible to distribute it to a relatively small number of students between lectures on a couple of occasions as multiple attempts would have affected the reliability of the results, through spill-over of the content and answers of CRT questions to other students. We did not include final year medical students because they were dispersed throughout the various teaching hospitals and were not accessible in a large group. There may be alternative reasons, other than better analytical skills, why some students scored higher System 2 responses than others. For instance, although we specifically asked students if they were previously aware of the CRT problems and excluded them if they answered in the affirmative, it is possible that some may have prior experience in similar kinds of mathematical problems and may therefore have found the CRT problems to be quite straightforward. This CRT test had only three questions; Frederick^27^ has used up to eight CRT problems in some studies, which may result in greater reliability. Ideally, we believe that the CRT test should have been followed up by a debrief where students could have explored the purpose of the test, the differences between Systems 1 and 2 thinking, the role of metacognition, and the importance of knowing how our minds think as novices, as experts, and in times of distress. Finally there is a need for ongoing research, including non-mathematical critical thinking tests, to assess the development of analytic and logical reasoning skills of medical students and emerging doctors over time.

The CRT mathematical test has shown that intuition is a dominant force in the minds of medical students. It has also shown that it is possible for this intuitive force to be put aside and for logic to prevail even as the CRT questions progress. Awareness and understanding of how experts think, in addition to intuition and metacognitive training, should be promoted amongst medical students as a way to aid their thinking processes and avoid cognitive errors in subsequent clinical practice. Finally, students need to understand how faulty or lazy thinking can lead to cognitive errors that can impact upon patient care and patient safety.

## Figures and Tables

**Table 1:** Responses to the CRT questions by pre-clinical students and students in clinical practice

	Correct Answer % (n)	System 1 intuitive or “impulsive” Answer % (n)	Incorrect Answer % (n)	Total
**Question 1**				
Pre-Clinical	65 (39)	30 (18)	5 (3)	100
Clinical	49 (33)	49 (33)	3 (2)	100
**Question 2**				
Pre-Clinical	73 (49)	15 (10)	12 (8)	100
Clinical	65 (40)	30 (18)	5 (3)	100
**Question 3**				
Pre-Clinical	84 (56)	10 (7)	6 (8)	100
Clinical	69 (42)	18 (11)	13 (8)	100
